# A New Small-Size Camera with Built-In Specific-Wavelength LED Lighting for Evaluating Chlorophyll Status of Fruit Trees

**DOI:** 10.3390/s23104636

**Published:** 2023-05-10

**Authors:** Xujun Ye, Marin Kitaya, Shiori Abe, Fanxing Sheng, Shuhuai Zhang

**Affiliations:** Faculty of Agriculture and Life Science, Hirosaki University, Aomori 036-8561, Japan

**Keywords:** low-cost and small-size camera, built-in LED, specific wavelengths, nutrient status evaluation, fruit trees

## Abstract

To produce high-quality crops, not only excellent cultivation techniques but also accurate nutrient management techniques are important. In recent years, many nondestructive tools such as the chlorophyll meter “SPAD” and the leaf nitrogen meter “Agri Expert CCN” have been developed for measuring crop leaf chlorophyll and nitrogen contents. However, such devices are still relatively expensive for individual farmers. In this research, we developed a low-cost and small-size camera with built-in LEDs of several specific wavelengths for evaluating the nutrient status of fruit trees. A total of 2 camera prototypes were developed by integrating 3 independent LEDs of specific wavelengths (Camera 1: 950 nm, 660 nm and 560 nm; Camera 2: 950 nm, 660 nm and 727 nm) into the device. In addition, a simple software tool was developed to enable the camera to capture leaf images under different LED lighting conditions. Using the prototypes, we acquired images of apple leaves and investigated the possibility of using the images to estimate the leaf nutrient status indicator SPAD (chlorophyll) and CCN (nitrogen) values obtained using the above-mentioned standard tools. The results indicate that the Camera 1 prototype is superior to the Camera 2 prototype and can potentially be applied to the evaluation of nutrient status in apple leaves.

## 1. Introduction

To produce high-quality apples to meet the rapidly growing market demand, key cultivation techniques such as artificial pollination, pruning of branches, thinning of flowers and fruits and bagging or rotating on-tree fruits have been adopted and widely practiced in Japan [1,2]. In recent decades, in addition to these meticulous cultivation techniques, optimal fertilization and nutrient management have attracted considerable attention in fruit production [3,4]. Precision nutrient management strategies have been proposed to replace conventional fertilizer practices for apple orchards [5,6]. Conventional fertilizing practices apply the same amount of fertilizer to the orchard regardless of the in-field variations in soil fertility and crop nutrient status, which raises the risk of overuse or underuse in different areas, resulting in less yield and fruit quality. In comparison, precision nutrient management strategies are characterized by site-specific and need-based nutrient management practices, which necessitate a thorough understanding of the nutrient status of the orchard on an individual tree basis [7,8]. It is therefore important to timely and accurately evaluate the nutrient status of individual trees in different growth stages when precision nutrient management strategies are implemented in orchards [9,10].

Chlorophyll is not only a pigment that gives the plant its green color, but is also an essential component for the conversion of light energy to stored chemical energy in the process of photosynthesis, which sustains plant life and promotes its growth. The chlorophyll pigment content in a leaf is directly related to the amount of solar radiation absorbed by the leaf, and eventually determines the photosynthetic capacity of the plant [11,12]. On the other hand, nitrogen is the most essential nutrient for crop production [13]. Particularly, in fruit production, it is directly involved in flower bud formation, flowering, fruit setting and development. For example, previous studies have found that the availability of nitrogen significantly influenced flowering intensity and the fruit set in olive trees [14]; higher nitrogen was correlated with budburst, flowering and fruit set, and improved the crop performance in peach trees [15]; and increasing nitrogen supply could lead to more cells per fruit, larger fruit and higher soluble solids in apple trees [16]. However, the excessive absorption of nitrogen in plants may lead to a deterioration in fruit quality, whereas a lack of nitrogen may cause significant yield reduction [17].

Traditionally, solvent-based leaf pigment extraction combined with spectrophotometric determination in solution [18] has been used to measure chlorophyll content, while the Kjeldahl method [19] and Dumas combustion digestion [20] have been used to quantify nitrogen concentration in plants. However, these methods depend on a destructive approach, and usually take a great deal of effort, time and money. Much research has demonstrated the potential of various optical sensors, reflectometers and image-based sensors in estimating these two indicators of crop nutrient status [10,21,22,23,24,25,26,27,28,29]. Numerous studies have investigated the relationships between the spectral characteristics of specific wavelengths and leaf chlorophyll/nitrogen contents, and based on which a variety of useful vegetation indices have been successfully developed for estimating the chlorophyll/nitrogen contents in crops [30,31]. Furthermore, based on thorough experimental investigation and evaluation, several portable electronic tools such as leaf chlorophyll meter “SPAD (Soil Plant Analysis Development)” (Konica Minolta, Inc., Tokyo, Japan), leaf nitrogen meter “Agri Expert CCN (Crop Control by Nitrogen)” (Satake Co., Ltd., Hiroshima, Japan), crop NDVI (normalized difference vegetation index) meters “GreenSeeker” (Trimble Navigation Limited, Westminster, CO, USA) and “Crop Circle” (Holland Scientific Inc., Lincoln, NE, USA), leaf color meter “Rice Scan” (Maxell Co., Tokyo, Japan), etc., have been developed and commercialized. These tools have been widely used as a nondestructive, rapid, accurate and effective method for estimating leaf nutrient status in both small-scale experiments and large-scale production fields [10,32,33,34,35,36,37].

During the past few decades, the significant relationships between chlorophyll and nitrogen contents in many agricultural and horticultural crops have been revealed by numerous studies. Although chlorophyll meters are designed to determine leaf chlorophyll concentration, the close relationships between chlorophyll and nitrogen contents in plant leaves have enabled these meters to be used as a standard tool for indirectly estimating leaf nitrogen in practical applications [38,39,40,41,42,43]. Furthermore, originally, these tools were developed for site-specific and need-based nutrient management in rice [44,45,46], and later they were also proven to be effective for evaluating nutrient status in many other crops [47,48,49,50,51,52]. However, such devices are still relatively expensive for individual farmers.

Our recent study has demonstrated the possibility of a multiple linear regression (MLR) model based on the raw reflectance of four narrowband specific wavelengths in estimating the nitrogen content in apple leaves [10]. Based on the above result, this research aimed to develop a low-cost and small-size camera for evaluating the nutrient status of fruit trees. In addition to the above-mentioned MLR model for nitrogen content estimation, this study first investigated the possibility of developing a similar MLR model for estimating chlorophyll content in apple leaves. Then, two camera prototypes were developed by incorporating the identified narrowband specific-wavelength LEDs into a “Rice Scan” device. A simple software tool was developed to control the camera and lighting system. The reflectance of the narrowband specific-wavelength LED light sources was extracted from the images acquired by the camera system. Finally, testing and evaluation experiments were carried out to assess the nutrient evaluation performance of the camera prototypes by means of relating the reflectance data to the leaf nutrient status indicators obtained by two widely used chlorophyll and nitrogen meters. The results demonstrated the potential applicability of the camera prototypes in estimating the nutrient status of apple leaves.

## 2. Materials and Methods

### 2.1. Selection of Key Wavelengths for Nutrient Status Evaluation

#### 2.1.1. Experiment

The experiments were carried out in the Fujisaki Farm of Hirosaki University, Japan (longitude 140°48′48.21″ E and latitude 40°65′61.6″ N). The most cultivated ‘Fuji’ apple trees (*Malus domestica*) grafted on the rootstock cultivar of ‘Marubakaidou’ (*Malus prunifolia* Borkh. Var. *ringo* Asami) at age 25 years were used in this study. The trees were planted at spacings of approximately 5 × 3.5 m and managed by the input of organic fertilizers made from apple leaves collected from the orchard. Apple leaves were collected from multiple trees in summer season (14 July 2020) and were screened to ensure a healthy, complete and undamaged status in the laboratory. In modeling analysis, because it is desirable to have a dataset with a wide distribution (variance) in the target variable (chlorophyll content), all of the leaf samples collected were first grouped via visual inspection of the leaf color, and then the leaf samples used for measurement were randomly selected from the groups. Therefore, the selected leaves exhibited large variation in degree of greenness. In the experiment, a total of 120 samples were prepared and measured within 4 h after sampling. 

An ImSpector V10E (Spectral Imaging Ltd., Oulu, Finland)-based hyperspectral imaging system [10] was used to obtain hyperspectral images for the collected leaf samples. During image acquisition, a white reference board was used to obtain the white reference, and the black reference was acquired when the camera shutter was completely turned off and the lens was covered by a black lens cap. The raw images were automatically calibrated based on the above references. This system collects hyperspectral images covering the 400–1000 nm spectral range with a spectral resolution of 5 nm, and each image has a spatial resolution of 1360 × 1024 pixels. With the Scanner Unit software (JFE Techno-Research Corporation, Tokyo, Japan) attached to the system, the average spectral reflectance data for each leaf sample were extracted and pretreated using a Savitzky–Golay smoothing algorithm for further analysis.

After the nondestructive hyperspectral image acquisition, the total chlorophyll concentration in leaf samples was determined using a conventional, destructive, solvent-based pigment-extraction method combined with an absorption spectrometer [10]. A total of 4 discs with 12 mm diameters cut from each leaf sample were prepared for pigment extraction using acetone solvent. After pigment extraction, the absorbances at 645 nm, 663 nm and 750 nm in the solvents were measured using a Genesys 10 Bio UV–VIS spectrophotometer (Thermo Fisher Scientific Inc., Waltham, MA, USA). Using the equation proposed by Mackenney [53], the amounts of total chlorophylls in each leaf sample were calculated.

#### 2.1.2. Data Analysis

During the analysis of hyperspectral data, it is always necessary to identify several so-called key wavelengths to reduce the high dimensionality of hyperspectral data. These key wavelengths can potentially be used to predict the chlorophyll content in the plant leaf. This is particularly important because the objective of this research is to develop a compact device with built-in specific-wavelength LEDs to measure the nutrient status of the plant leaf, and such a small device could not provide enough space to accommodate too many LEDs. 

A few statistical methods have been proposed for selecting the key wavelengths from high-dimensional spectral data [10]. This study employed Pearson’s correlation analysis [54] to identify the key wavelengths. The correlation coefficients between the reflectance of specific wavelengths and chlorophyll content were used as a criterion. The wavelengths whose reflectance had the highest correlations with chlorophyll content in certain wavelength ranges were selected. These key wavelengths were then used to develop a chlorophyll content prediction model using a multiple linear regression (MLR) algorithm. In modeling, the sample data were divided into 2 subsets: 2/3 of the entire data (80 samples) was used as the training set to develop the model and the remaining 1/3 of the entire data (40 samples) was used as the test set to evaluate the model’s performance. Finally, the performance of the MLR model was used to assess the usefulness of the selected key wavelengths for evaluating the nutrient status of the plant leaf.

### 2.2. Design and Development of Camera Prototype and Software

The ultimate purpose of this research was to develop a low-cost and small-size camera for evaluating the nutrient status of fruit trees. The camera was expected to be able to capture the images of fruit leaf samples under narrowband light sources such as LEDs. The first prototype version of “Rice Scan” (Maxell Co., Tokyo, Japan) was used as the basis for the design and development of the camera prototype. “Rice Scan” is a small RGB camera developed for assessing the nutrient status in rice crops based on a leaf color index. The camera has been tested and evaluated across Japan, with varying degrees of success, and it became available in the market in 2018. However, shortly after the release of the product, due to certain reasons, the product development and services were terminated. In this study, a new camera prototype was equipped with multiple narrowband LED light sources instead of a single broadband white LED light source in “Rice Scan”. These built-in LEDs emitted the narrowband specific-wavelength lights of the key wavelengths that were identified in the preceding experimental research. 

To control the camera prototype, a simple software tool was designed and developed under the Visual Studio IDE environment (Microsoft Corporation, Redmond, WA, USA). With the help of the software, the camera prototype could be easily triggered to acquire images of leaf samples under each narrowband specific-wavelength LED light condition, and the images could be saved in a computer for further analysis.

### 2.3. Testing and Evaluation of Camera Prototype

To investigate the feasibility of using the camera prototype to assess the nutrient status of the apple leaf, the following experiments and data analyses were performed: firstly, the chlorophyll meter “SPAD-502plus” (Konika Minolta, Inc., Tokyo, Japan) and the leaf nitrogen meter “Agri Expert CCN6001” (Satake Co., Ltd., Hiroshima, Japan) were employed to measure the SPAD (index value, a relative indicator of chlorophyll content calculated based on the absorbances of the wavelengths 650 nm and 940 nm) and CCN (nitrogen content in % calculated based on the absorbances of 4 unpublished wavelengths) values of leaf samples; secondly, the camera prototype was used to capture the images of leaf samples under the identified narrowband specific-wavelength LED light conditions; thirdly, data extraction from the images was performed and the extracted data were then related to the SPAD and CCN values for the same samples by means of multiple linear regression analysis. 

During the process of data analysis, the RGB data were first extracted from the images captured using the two camera prototypes. Because the prediction models for chlorophyll and nitrogen contents were developed based on the reflectance of key wavelengths, the reflectance intensity of specific-wavelength light emitted from the built-in LEDs needed to be calculated from the raw RGB data. To obtain the reflectance intensity of the specific-wavelength LED emissions, the raw RGB data were converted to L*a*b* values. In an L*a*b* color space, the L* value is a measurement of the lightness or amount of light reflected; the a* value indicates the intensity of the red (+ values) or green (− values) color and the b* value indicates the intensity of the yellow (+ values) or blue (− values) color [55]. Therefore, the L* value represents the reflectance intensity of a specific-wavelength LED light, and can potentially be used to predict chlorophyll and nitrogen contents with the prediction models developed. 

In this study, we investigated the possibility of using the data captured using the camera prototypes to predict the SPAD and CCN values. The L* values of the specific-wavelength LEDs obtained for each prototype were used to develop multiple linear models for predicting the SPAD and CCN values. Considering the leaf color values obtained under the specific-wavelength LED illuminations may provide valuable nutrient-related information; the a* and b* values in addition to the L* values and the raw RGB values were also used to develop prediction models.

Because the commercial “SPAD” and “Agri Expert CCN” meters have been widely used in agricultural fields as the nondestructive standard tools to evaluate the nutrient status of plant leaves, a high correlation between the data obtained by the prototype and the SPAD and CCN values would confirm the possibility of the prototype being used as an alternative to these two standard tools.

## 3. Results and Discussion

### 3.1. Characteristics of Reflectance Spectra

**Figure 1:**
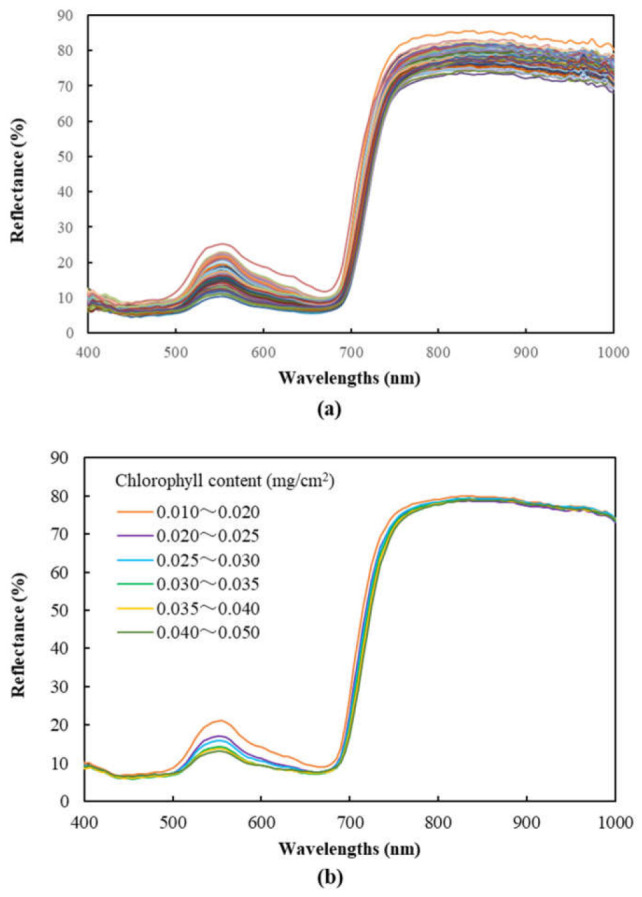
Spectral reflectance characteristics of ‘Fuji’ apple leaf samples: (**a**) all individual leaf samples (each line indicates the reflectance spectrum of an individual leaf sample); (**b**) the average of leaf sample groups with different levels of chlorophyll content.

Figure 1 shows the reflectance spectra obtained in the experiment. Significant differences in the reflectance intensity of certain wavelengths, particularly those in the green to red, the red edge and the near infrared wavelength ranges were found among the leaf samples (Figure 1a). Leaf samples were grouped with different levels of chlorophyll content, and the average reflectance of the groups was calculated (Figure 1b). It was found that as the levels of chlorophyll content increased, the group average reflectance spectra shifted downward sequentially, forming an orderly distribution of group spectra. Particularly, the wavelengths in the green to red and the red edge spectral ranges demonstrated larger reflectance intensity differences among different groups. This suggests that these wavelength ranges may be more closely associated with the chlorophyll content in apple leaves than other wavelengths. Our previous research also showed the similar relationship between reflectance intensity and nitrogen content in apple leaves and confirmed the usefulness of several key wavelengths in these ranges in estimating the nitrogen content in apple leaves [10].

### 3.2. Correlation between Spectral Reflectance and Chlorophyll Content

**Figure 2:**
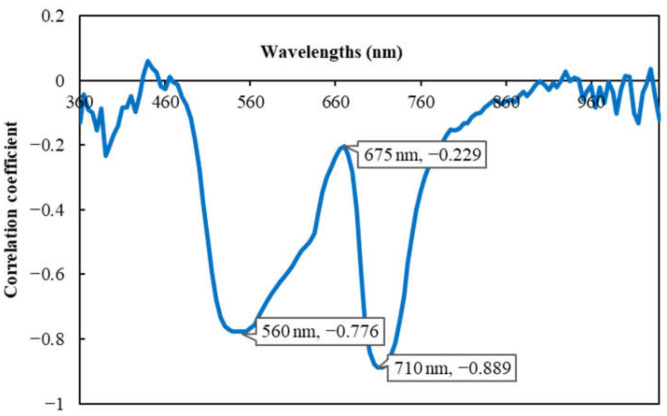
Correlation coefficients between ‘Fuji’ apple leaf reflectance at each wavelength and chlorophyll content. For all of the wavelengths, 3 characteristic wavelengths (560 nm, 710 nm and 675 nm) and their correlation coefficients are indicated in the graph: the wavelengths (560 nm and 710 nm) show the highest negative correlation with chlorophyll content and the characteristic wavelength (675 nm) represents a peak between the above 2 wavelengths.

Pearson’s correlation coefficients between leaf chlorophyll content and the reflectance of each wavelength are illustrated in Figure 2. The reflectance of most wavelengths individually showed a negative correlation with chlorophyll content. Among them, the wavelengths 710 nm and 560 nm demonstrated the highest negative correlations, achieving a significant correlation coefficient of −0.889 and −0.776 (*p* < 0.001), respectively. The wavelength 675 nm, whose correlation coefficient with chlorophyll content was the peak but the lowest in the range between the above 2 wavelengths (*r* = −0.229, *p* < 0.001), could be recognized as a characteristic wavelength because it possesses a significant feature in spectral reflectance compared to its longer or shorter adjacent wavelengths. Our previous study [10] revealed that the reflectance of the wavelengths around 560 nm (green) and 710 nm (red edge) was directly associated with leaf nitrogen content, while the wavelength at 675 nm (red) showed a significant correlation with leaf nitrogen content in terms of its reflectance’s first derivatives (the rates of reflectance increased). Therefore, the three wavelengths were almost the same wavelengths as those found in our previous study [10]. Numerous previous studies have confirmed the significant relationships between chlorophyll content and nitrogen content in plant leaves [10,24,38,39]; therefore, the above 3 specific wavelengths (560 nm, 675 nm and 710 nm) could potentially be used as the key wavelengths to estimate both chlorophyll and nitrogen contents in apple leaves.

### 3.3. Chlorophyll Content Prediction Model

**Figure 3:**
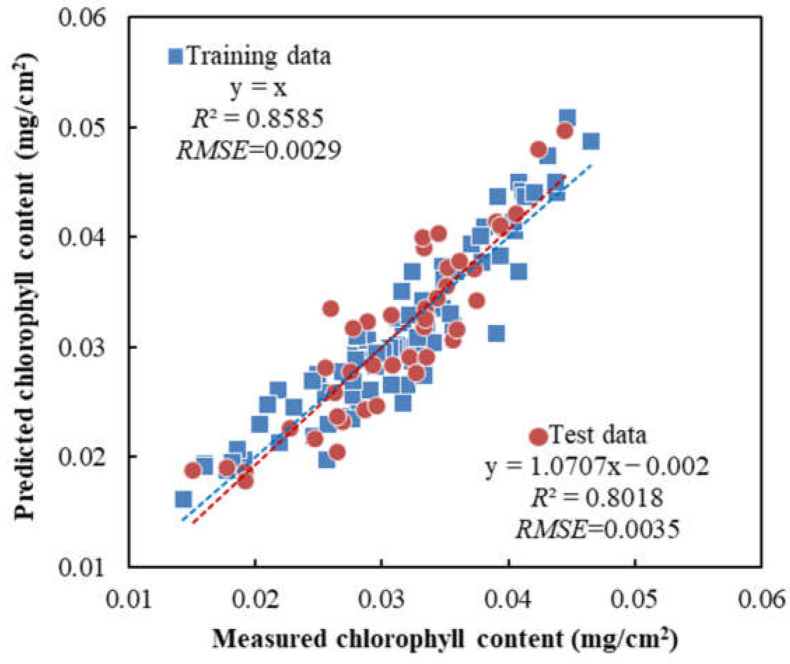
Measured vs. predicted chlorophyll content in ‘Fuji’ apple leaf samples via the MLR model based on the three key wavelengths. The dashed lines indicate the best fit lines for the training data set (blue) and the test data set (red), respectively.

Based on the above analysis, 3 key wavelengths, i.e., 560 nm, 675 nm and 710 nm, were identified and used to develop an MLR model for chlorophyll content prediction. Figure 3 illustrates the performance of the MLR model for the prediction with both the training and the test data sets. The model achieved high predictive accuracy in the predictions with both the training (*R*^2^ = 0.8585 and *RMSE* = 0.0029 (*p* < 0.001)) and the test (*R*^2^ = 0.8018 and *RMSE* = 0.0035 (*p* < 0.001)) data sets, showing high consistency in the model predictions. This result confirmed the usefulness of the above three key wavelengths for estimating the chlorophyll content in apple leaves. These key wavelengths are among the key wavelengths that were selected for estimating the nitrogen content in apple leaves from earlier research [10].

### 3.4. Design and Development of Camera Prototype

**Figure 4:**
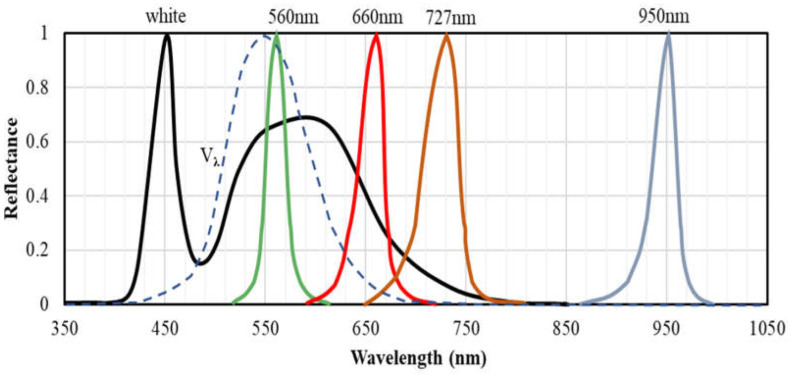
Relative spectral emission of the 4 specific-wavelength LEDs (LP M675 (560 nm) (green line), GH DASPA2.24 (660 nm) (red line), GF DASPA2.24 (727 nm) (orange line), and SFH4646 (950 nm)) (grey line). The emission spectrum of the white LED used in “Rice Scan” is indicated by black solid line. *V_λ_* is the CIE (International Commission on Illumination) standard photopic luminous efficiency function (blue dashed line). This figure was produced based on the datasheet of each specific-wavelength LED product and the measured emission spectrum of the white LED embedded in “Rice Scan”.

In designing the camera prototype, three LEDs, each of which emitting light at one of the three key wavelengths, were used as the lighting for the prototype. In addition, another LED at the wavelength of 950 nm was added into the prototype, which, due to its high reflectance, has always been used as a reference in the development of similar devices [56,57]. Since the LEDs 675 nm and 710 nm were not available in the market, it was recommended to replace them with the LEDs 660 nm and 727 nm that had the closest wavelengths to each of the 2 key wavelengths available in the market. Finally, the commercial narrow-band LEDs manufactured by arm-OSRAM (OSRAM GmbH, München, Germany), LP M675 (560 nm), GH DASPA2.24 (660 nm), GF DASPA2.24 (727 nm) and SFH4646 (950 nm) were purchased for the prototype development. The relative spectral emissions of the four LEDs are shown in Figure 4. Each LED emits a light with the peak at/near the key wavelength and a narrow full width at half maximum (FWHM). The emission spectrum of the white LED used in “Rice Scan” (black solid line) and the CIE (International Commission on Illumination) standard photopic luminous efficiency function *V_λ_* (dashed line) are indicated for comparison.

**Figure 5:**
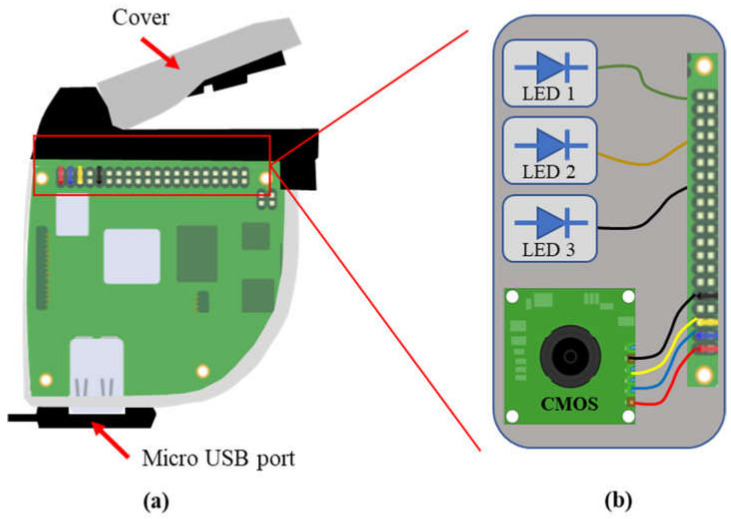
Diagrammatic representation of the camera: (**a**) the whole device; (**b**) the enlarged part of the lighting and sensor system (3 LEDs and 1 CMOS sensor).

Due to limited space in the small prototype designed, only three LEDs could be added into the prototype. Therefore, two camera prototypes were developed separately, each with a different combination of three LEDs (Table 1). Figure 5 shows the diagrammatic representation of the camera prototypes. The lighting system including three LEDs and a CMOS sensor (OMNIVISION OV7739) were integrated on the upper side of the board. A micro-USB port was designed on the lower part of the prototype.

**Figure 6:**
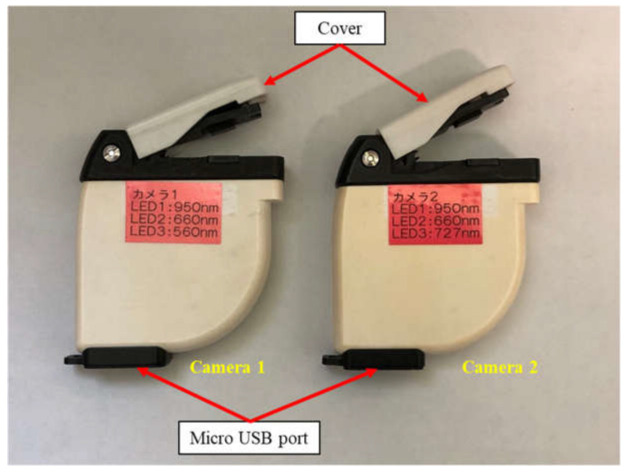
The two camera prototypes developed with different combinations of three specific-wavelength LEDs. Left: Camera 1 (950 nm, 660 nm and 560 nm); right: Camera 2 (950 nm, 660 nm and 727 nm). The Japanese syllabary characters “カメラ” on the camera prototypes mean “camera” in English.

**Figure 7:**
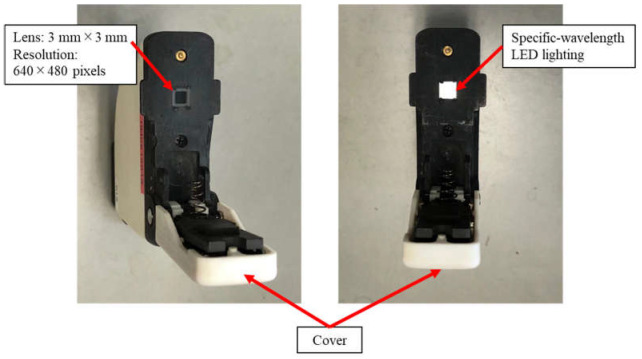
The camera prototype is equipped with an entrance window of 3 mm × 3 mm and a CMOS sensor that can capture an RGB image with a resolution of 640 × 480 pixels. During measurement, a plant leaf was placed on the entrance window and the cover was put down closely on the leaf to avoid ambient light interference. A specific-wavelength LED was selected with the software, and the image was then captured under the emitted light condition.

The two camera prototypes developed are shown in Figure 6 and Figure 7. The prototype is a palm-sized (70 mm height × 60 mm width × 18 mm depth) and low-weight (60 g) device, equipped with an entrance window of 3 mm × 3 mm and a CMOS sensor that can capture an RGB image with a resolution of 640 × 480 pixels for an area of 2 mm × 2 mm on the leaf sample. During measurement, a plant leaf was placed on the entrance window and the cover was put down closely on the leaf to eliminate the ambient light interference. A specific-wavelength LED was selected with the software developed in parallel, and the image was then captured under the emitted light condition.

### 3.5. Design and Development of Software for Camera Prototype

**Figure 8:**
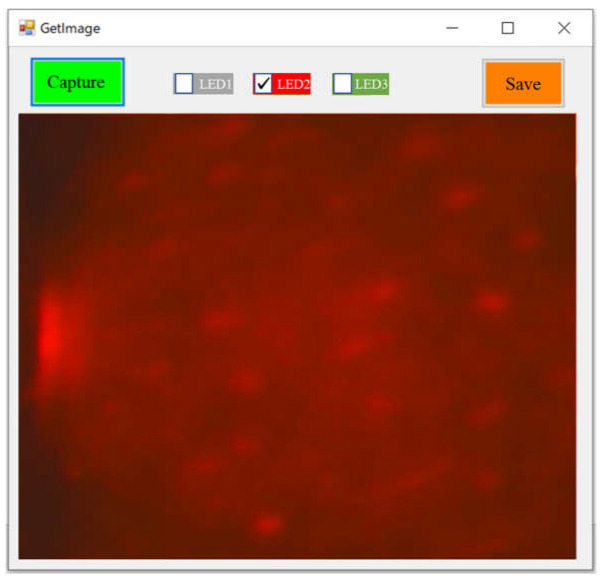
The interface of the software developed to capture images for the camera prototype.

A software tool for controlling the camera prototype was designed and developed under the Visual Studio IDE environment (Microsoft Corporation, Redmond, WA, USA). Figure 8 shows the interface of the software to capture images using the camera prototype. In image acquisition, a specific-wavelength LED was first selected, and the light of the selected specific wavelength was emitted when the image was captured using clicking the “Capture” button. The captured image could be saved into a designated folder by clicking the “Save” button. The image shown in the figure was captured for a green leaf under the LED2 (660 nm) lighting condition using the Camera 1 prototype.

**Figure 9:**
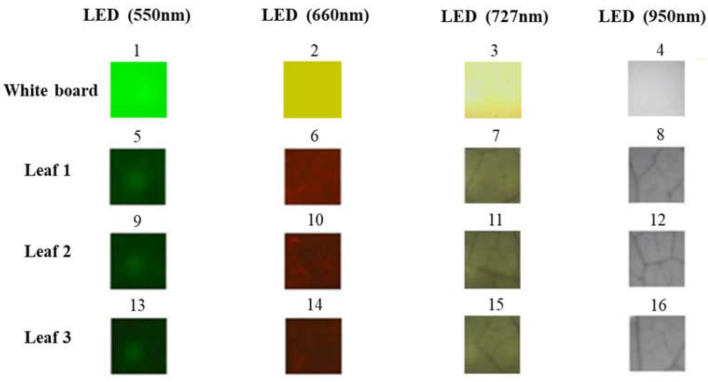
RGB images for the white board and three leaf samples captured using the camera prototypes under four different specific-wavelength LED lighting conditions. The numbers indicate the number of images taken sequentially with the cameras.

Figure 9 shows the images captured for the white board (used as a reference) and three apple leaf samples using the two camera prototypes. The images captured under the four LED lighting conditions show significant differences between one another. This suggests the possibility of extracting the reflectance of specific-wavelength light emitted from the built-in LEDs on the leaf from the images, which can be used for estimating the nutrient status of the leaf.

### 3.6. Relationships between SPAD, CCN Value and Chlorophyll Content

**Figure 10:**
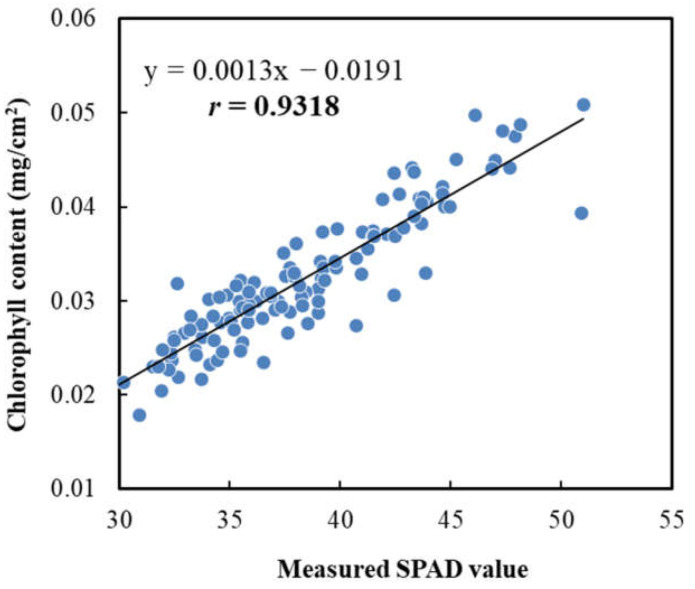
Correlation between measured SPAD values and chlorophyll content in ‘Fuji’ apple leaves. The line indicates the best fit line.

The relationships between the nondestructively measured SPAD and CCN values, and between the SPAD value and the chlorophyll content measured using a destructive, solvent-based pigment-extraction method combined with an absorption spectrometer were investigated. A significant correlation (*r* = 0.9318 (*p* < 0.001)) between the measured SPAD values and chlorophyll contents in leaves was found (Figure 10), suggesting the usefulness of the SPAD meter in evaluating the chlorophyll content in the apple leaf. This agrees with the results of numerous previous studies that have confirmed the effectiveness of using SPAD values to assess the nutrient status of a variety of plants and crops [42,43,44,50,51,52].

**Figure 11:**
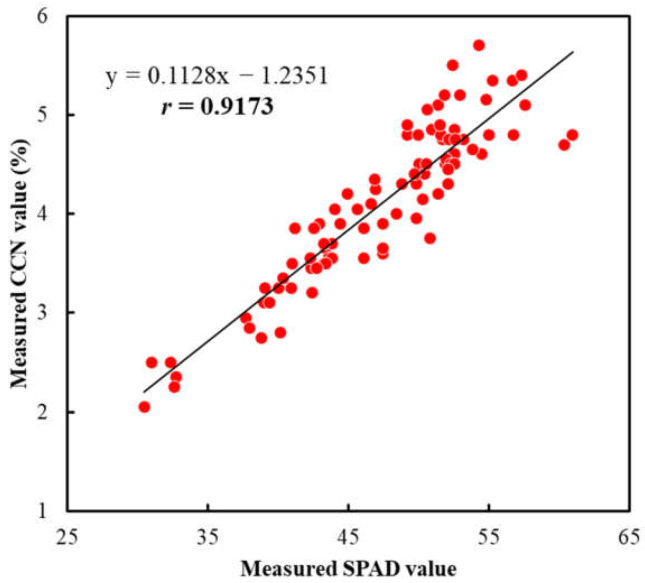
Correlation between measured SPAD values and measured CCN values for ‘Fuji’ apple leaves. The line indicates the best fit line.

In addition, there was also a significant correlation (*r* = 0.9173 (*p* < 0.001)) between the measured SPAD and CCN values (Figure 11). Although the “SPAD meter” and “Agri Expert CCN” meter were developed for measuring the chlorophyll and nitrogen contents in the leaf plant, respectively, the results measured using these two devices were significantly correlated with each other. Similar results have also been reported in the literature and the reasons have mainly been attributed to the close relationship between the chlorophyll and nitrogen contents in the plant leaf [10,24,38,39]. Our previous study confirmed that the SPAD value not only could be used to accurately predict the chlorophyll content, but it could also be used to predict the nitrogen content in the apple leaf with reasonable accuracy [47]. Furthermore, the CCN value could be directly interpreted into the actual level of nitrogen concentration in the plant leaf [58,59,60]. Therefore, if the camera prototype developed in this research could accurately predict the SPAD and CCN values, it could potentially be used as a new tool to evaluate the nutrient status of plants and crops.

### 3.7. Performance of SPAD and CCN Prediction Models Based on Images Captured Using Camera Prototype

**Figure 12:**
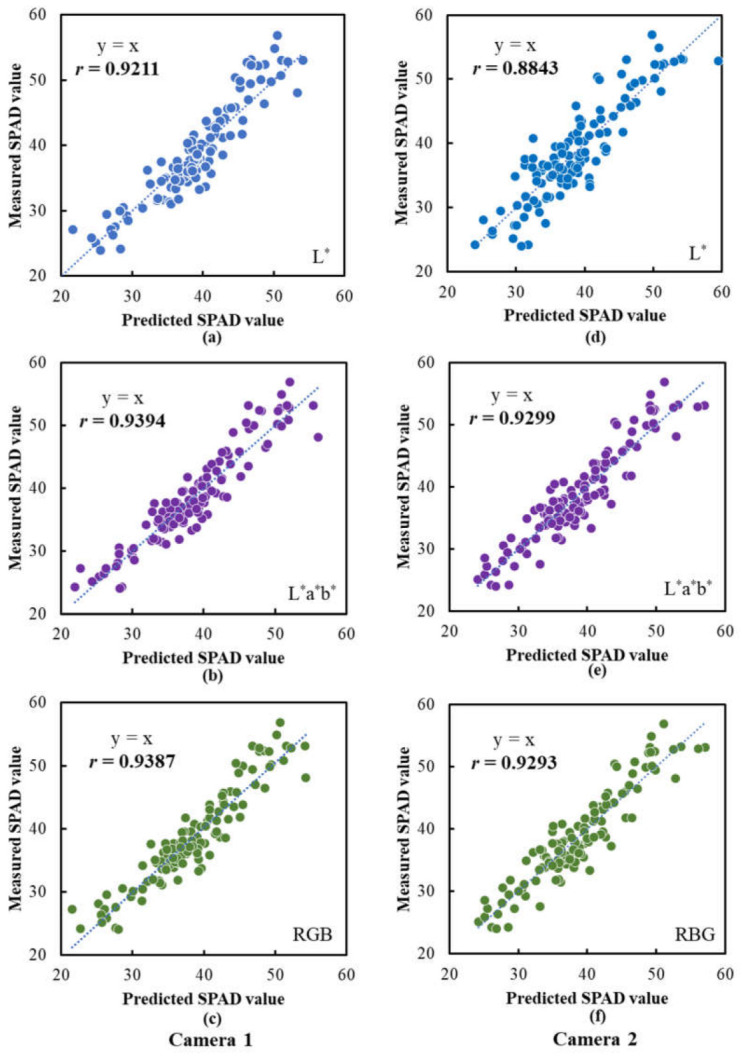
Performances of the SPAD prediction models based on the data extracted from the images captured using the camera prototypes. Scatter plots of the measured SPAD values vs. the predicted SPAD values are illustrated: (**a**–**c**) are the results for the models based on L*, L*a*b* and RGB data, respectively, for Camera 1; (**d**–**f**) are the results for the models based on L*, L*a*b* and RGB data, respectively, for Camera 2. The lines indicate the best fit lines.

The SPAD and CCN values for leaf samples predicted using the above models were plotted against the measured SPAD and CCN values, and simple correlation analyses were conducted to investigate the relationships between them. Figure 12 shows the scatter plots of the measured SPAD values vs. the predicted SPAD values. For Camera 1, the models based on the L*, L*a*b* and RGB data all performed excellently in predicting the SPAD values, with correlation coefficients being 0.9211, 0.9394 and 0.9387, respectively. In comparison, for Camera 2, the model based on the L* data obtained a correlation coefficient of 0.8834, which is significantly lower than the same model for Camera 1, and the models based on the L*a*b* and RGB data also performed well with predictive accuracies that were only slightly lower than those for Camera 1. These results indicated that, overall, Camera 1 was superior to Camera 2 in predicting the SPAD values, and the inclusion of color information in addition to the specific-wavelength reflectance intensities could improve the performance of the prediction models.

**Figure 13:**
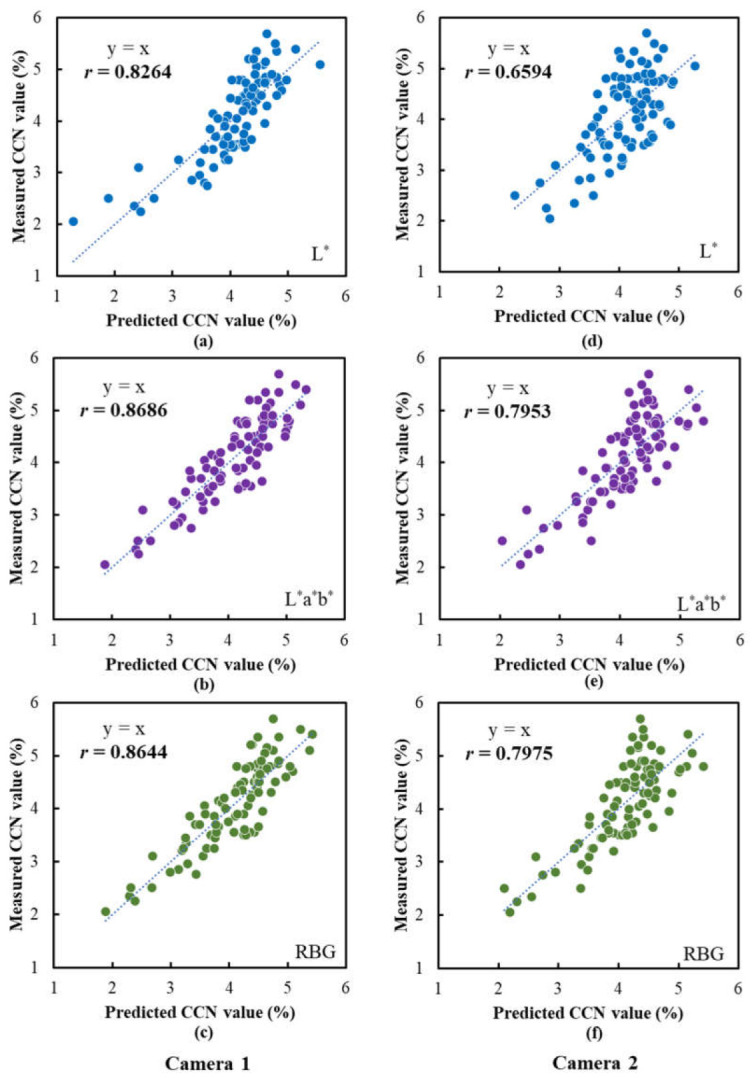
Performances of the CCN prediction models based on the data extracted from the images captured using the camera prototypes. Scatter plots of the measured CCN values vs. the predicted CCN values are illustrated: (**a**–**c**) are the results for the models based on L*, L*a*b* and RGB data, respectively, for Camera 1; (**d**–**f**) are the results for the models based on L*, L*a*b* and RGB data, respectively, for Camera 2. The lines indicate the best fit lines.

Figure 13 shows the scatter plots of the measured CCN values vs. the predicted CCN values. Compared to the models for the SPAD values, the models for the CCN values all achieved a relatively lower prediction accuracy. For Camera 1, the correlation coefficients between the measured and the predicted CCN values for the models based on the L*, L*a*b* and RGB were 0.8264, 0.8686 and 0.8644, respectively. Furthermore, the models for Camera 2 performed worse than the corresponding models for Camera 1. Among them, the model based on the L* data obtained the lowest correlation coefficient of 0.6594, and the models based on the L*a*b* and RGB data did not perform satisfactorily, with the predictive accuracies being significantly lower than those for Camera 1. These results indicated that, similarly, Camera 1 was superior to Camera 2 in predicting the CCN values, and the usefulness of color information in improving the performance of prediction models was confirmed. The above results suggest that the images captured using the Camera 1 prototype may contain more nutrient-related information on “Fuji” apple leaves than those captured using the Camera 2 prototype.

The above findings demonstrated the potential usefulness of the camera prototypes in evaluating the nutrient status in the apple leaf. Particularly, Camera 1 had a superiority over Camera 2 in predicting both the SPAD values and the CCN values, showing a more potential applicability for real practices in the future. This study serves as a preliminary step toward developing a low-cost, small-size and easy-to-use tool for the evaluation of nutrient status in plants and crops. The new tool is expected to be used as an alternative to the existing commercial tools. Compared to the tools currently available in the market (such as “SPAD”, “GreenSeeker” and “Agri Expert CCN” meters), whose prices range from USD 800 to 2000, the total cost for each camera prototype developed in this study was only between USD 100 and 200. Such a low-cost tool will be very attractive to farmers. Future work will include the development of new versions of the camera prototypes with built-in Wi-Fi capability as well as the development of a mobile application for mobile phones and devices to remotely control the camera. Wi-Fi capability will enable the wireless mobility of the camera, and the new mobile app system is expected to be able to quickly calculate SPAD, CCN and chlorophyll/nitrogen content estimates based on the images captured using the camera, and will show the results on mobile devices in real time. These new features will further increase the usability of the tool in crop nutrient management in the future.

## 4. Conclusions

Our recent study revealed the possibility of using a simple MLR model based on four narrowband specific wavelengths to estimate the nitrogen content in apple leaves. With an aim to apply the above model in a practical situation, the present research developed a low-cost and small-size camera that could collect narrowband specific wavelength reflectance for evaluating the nutrient status of fruit trees. Two camera prototypes were developed by integrating three independent LEDs of specific wavelengths into a “Rice Scan” device. Using the camera prototypes, we acquired images of ‘Fuji’ apple leaves and investigated the possibility of using the images to estimate the nutrient status indicators obtained by the widely used “SPAD” and “Agri Expert CCN” devices. The results indicated that the data obtained by the Camera 1 prototype had higher correlations with the nutrient status indicators than those obtained by the Camera 2 prototype. This study suggests that the new low-cost and small-size camera with built-in specific-wavelength LED lighting developed in this study can potentially be applied to the evaluation of nutrient status in apple leaves.

## Figures and Tables

**Table 1:** Narrowband specific-wavelength LEDs used in the two camera prototypes.

LED No.	Camera Prototype 1		Camera Prototype 2	
	λ (nm)	Product No.	λ (nm)	Product No.
LED 1	950	SFH4646	950	SFH4646
LED 2	660	GH DASPA2.24	660	GH DASPA2.24
LED 3	560	LP M675	727	GF DASPA2.24

*λ*: narrowband specific wavelength; all LEDs were manufactured by arm-OSRAM (OSRAM GmbH, München, Germany).

## Data Availability

Not applicable.
